# New Delivery Systems of Stem Cells for Vascular Regeneration in Ischemia

**DOI:** 10.3389/fcvm.2017.00007

**Published:** 2017-02-24

**Authors:** Adegbenro Omotuyi John Fakoya

**Affiliations:** ^1^Department of Anatomical Sciences, All Saints University School of Medicine, Roseau, Dominica

**Keywords:** ischemia, stem cells, delivery, growth factors, angiogenesis, transplantation, macrophages

## Abstract

The finances of patients and countries are increasingly overwhelmed with the plague of cardiovascular diseases as a result of having to chronically manage the associated complications of ischemia such as heart failures, neurological deficits, chronic limb ulcers, gangrenes, and amputations. Hence, scientific research has sought for alternate therapies since pharmacological and surgical treatments have fallen below expectations in providing the desired quality of life. The advent of stem cells research has raised expectations with respect to vascular regeneration and tissue remodeling, hence assuring the patients of the possibility of an improved quality of life. However, these supposed encouraging results have been short-lived as the retention, survival, and engraftment rates of these cells appear to be inadequate; hence, the long-term beneficial effects of these cells cannot be ascertained. These drawbacks have led to the relentless research into better ways to deliver stem cells or angiogenic factors (which mobilize stem cells) to the regions of interest to facilitate increased retention, survival, engraftment, and regeneration. This review considered methods, such as the use of scaffolds, retrograde coronary delivery, improved combinations, stem cell pretreatment, preconditioning, stem cell exosomes, mannitol, magnet, and ultrasound-enhanced delivery, homing techniques, and stem cell modulation. Furthermore, the study appraised the possibility of a combination therapy of stem cells and macrophages, considering the enormous role macrophages play in repair, remodeling, and angiogenesis.

## Introduction

Ischemia is a partial or complete interruption of blood supply to a tissue or organ with an accompanying interruption of the flow of oxygen, glucose, and tissue washout resulting in tissue death by necrosis. Most pertinent to the medical practice are cardiovascular diseases (CVDs), a disease of the heart and blood vessels of which myocardial infarction (MI), stroke, and peripheral arterial diseases (PAD) is the most notable. More than 27 million Americans (1) and 202 million people worldwide (2) have experienced an ischemic heart event, stroke, or hospitalization for heart failure, peripheral arterial disease with smoking, increasing age, and diabetes being major risk factors (2). The American Heart Association estimates the annual cost of CVD to be $155 billion in direct health-care costs and another $92 billion in indirect costs reflecting lost productivity (3). CVDs, mainly acute MI, caused by the occlusion of the coronary arteries are the leading cause of mortality worldwide (4, 5). CVD usually damages the heart muscle cells [cardiomyocytes (CMCs)] irreversibly resulting in complications associated with MI such as early mortality occurring from arrhythmias such as ventricular tachycardias and fibrillations (6, 7). CMCs have previously been regarded as post-mitotic cells; however, increasing research evidence has disproved this with observations revealing that ischemic events may influence the proliferative capacity of adult mammalian CMCs (8), though young adult CMCs have an annual renewal rate of about 1% (9). The world heart federation, 2016, stated that stroke which occurs as a result of the loss of blood supply to the brain is the seconding leading cause of mortality over 60 years of age and the fifth between the ages of 15 and 59 years resulting in a long-term neurological deficit. Another debilitating ischemia is that of the lower limb, which may lead to gangrene and ultimately amputation of the affected limb. Lower limb ischemia can either be an acute (as a result of an arterial embolism) or a critical limb ischemia (which is an end-stage result of a PAD) affecting about 2 million Americans (1, 10). Atherosclerosis, an inflammatory disease, is the primary pathophysiology underlying CVDs in which endothelial dysfunction is a critical initial event in its pathogenesis, and this contributes to the initiation and progression of plaque (11, 12). Research has been carried out on diverse stem cells with rather promising results. However, quite a few challenges still need to be overcome, such as identifying the most appropriate mode of delivery, timing of delivery, survival, retention, and integration of these therapeutic cells to the site of ischemia with enhancement of revascularization and angiogenesis. The goal of a delivery system is to be able to transplant the right amount of (stem) cells to the site of interest and attain the maximal cell retention and ultimately prevent the deleterious effect of the ischemia such as amputation, heart failure, and neurological deficits. Thus, this study examined previous methods of stem cell delivery and the new methods being used to deliver stem cells for an efficient vascular regeneration.

## Methods

Data for this study were obtained from Medline on OvidSP, which includes PubMed, Embase by the US National Library of Medicine as well as a search through the University of Bristol Library services.

### Search Strategy

The search was carried out by signing into Ovid, Wolters, and Kluver portal and “All Resources” was selected. The keyword “stem cell delivery” was used with a further selection made on stem cell transplantation. So search for stem cell delivery/stem cell transplantation gave a total number of 19,919 publications. The second search with the keyword “ischemia/tissue ischemia” yielded a total number of 46,597 articles.

Combining the search for “stem cell delivery/stem cell transplantation” using the Boolean operator “AND” with “ischemia/tissue ischemia” yielded a total of 274 publications. Publications were then further hand screened to ascertain if they fit into the inclusion the criteria for the study and I arrived at a total of about 66.

However, other data included in the release were obtained from the University library services by simply using the search phrase “stem cell delivery” and “stem cells in ischemia” and articles around vasculogenesis/angiogenesis and were hand screened to fit the inclusion criteria and 38 other publications were selected. Also included were relevant references from previously selected articles as well as some recommended publications. A total of 171 articles were reviewed.

### Inclusion Criteria

Publications selected were analyzed thoroughly to see if they focused on the study which was on delivery of stem cells in the setting of ischemia. Included were studies that utilized stem cells (such as embryonic, mesenchymal, adult, endothelial progenitor, cardiac, hematopoietic, and induced pluripotent stem cells) in the setting of ischemia such as myocardial ischemia, limb ischemia, and cerebral ischemia. Also considered were publications which included scaffolds in the delivery of stem cells and new modes of targeting and delivering stem cells to sites of ischemia and articles that also considered tracking of these cells to target sites.

## Brief Overview of the Molecular Basis of Blood Vessel Formation

Vasculogenesis and angiogenesis are the two methods for blood vessel formation. The formation of primordial blood vessels is known as vasculogenesis, and this occurs during embryogenesis as a result of increasing metabolic and oxygen demands of the organs of the embryo as their sizes increases. Four major steps are involved in this, and this includes (i) mesoderm formation, (ii) differentiation into blood islands, (iii) blood island fusion, and (iv) primary capillary plexus formation. Nevertheless, new sites of vascularizations have been demonstrated in adults as well (13). Angiogenesis, however, is the formation of new blood vessels from previously existing ones, and two processes are involved in this (i) budding/sprouting, this is the growth phase, where stabilization of new blood vessels occurs and (ii) intussusception, here the local vascular network is remodeled by the insertion of interstitial cells which helps to partition the new vessels (14).

**Figure 1:**
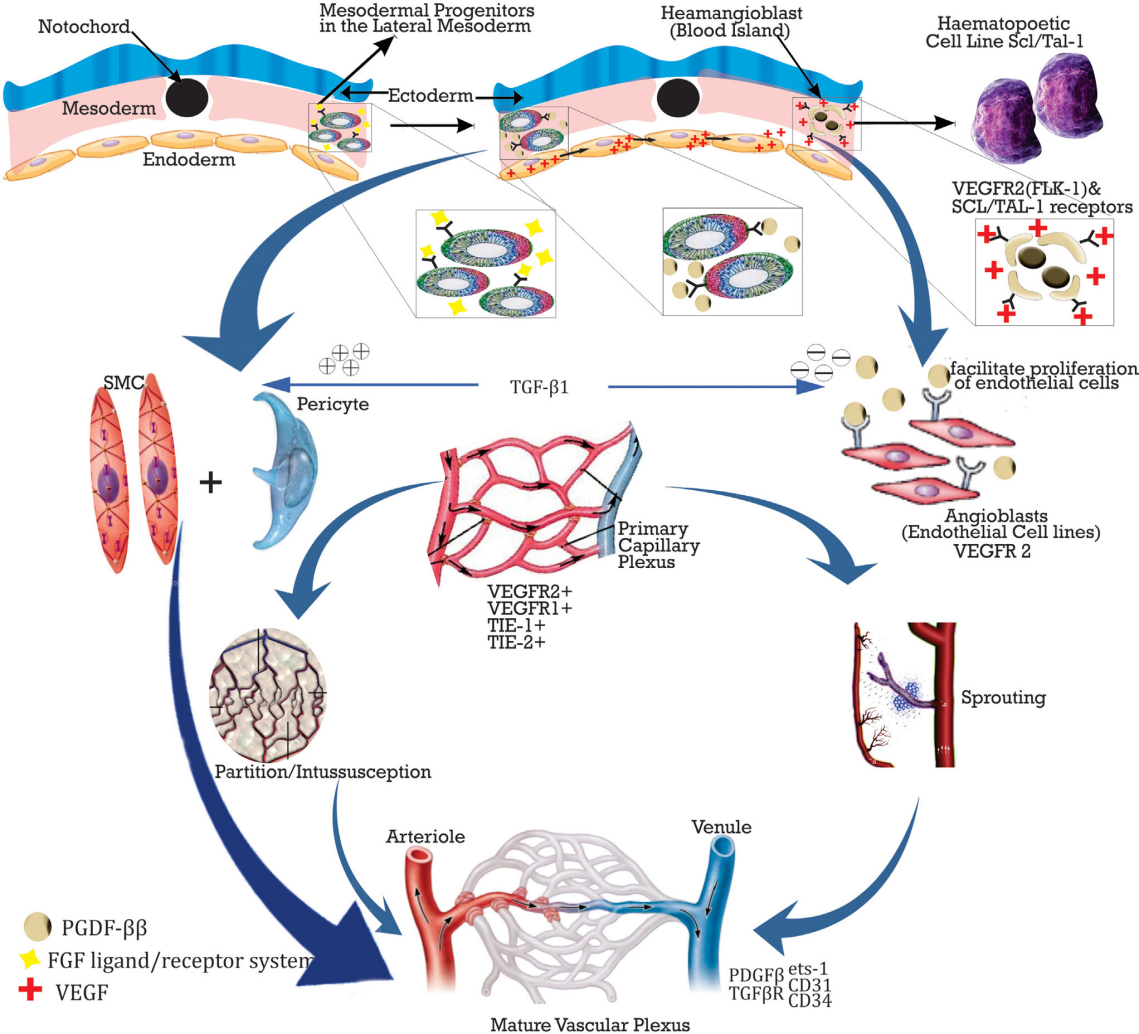
**Summary of vasculogenesis and angiogenesis with some molecular overview**.

Vasculogenesis is initiated in the embryo by the fibroblast growth factor (FGF) ligand/receptor system inducing the mesoderm to form blood islands. The endoderm then releases the vascular endothelial growth factor (VEGF) following the expression of its receptors VEGF-R2 (Flk-1) and SCL/TAL-1 by haemangioblasts thus facilitating its differentiation into hematopoietic and endothelial cell (EC) lines. Another receptor, VEGF-R1 (Flt-1) is also released but however utilized late in angiogenesis to promote aggregation of angioblasts. VEGF facilitates differentiation into ECs, while platelet-derived growth factor (PDGF)-BB induces smooth muscle (SMC) formation and is mitogen and for both SMCs and pericytes. Angiopoietin (Ang-1), a Tie-1(tek) ligand enhances luminal formation and stabilization, while Ang-2 halts the process. EC proliferation is then enhanced by PDGF-BB bound to receptor PDGFBR and transforming growth factor (TGF)-B1 antagonizes this by facilitating the contact between ECs and pericytes (14). Other factors involved in adhesion of ECs and junction formation include endothelial transcriptase (ets)-1, platelet endothelial cell adhesion molecule (PECAM)-1 (CD31), vascular endothelial-cadherin, and CD34 (15). Figure 1 below shows the schematic representation of the process of vasculogenes.

Angiogenesis on the hand involves (i) migration of ECs, (ii) tubulogenesis, (iii) vessel maturation, and (iv) vessel stabilization. The expression of either VEGF or hepatocyte growth factor (HGF), which initiates angiogenesis facilitates this process by subtly different mechanisms.

Vascular endothelial growth factor, when expressed, increases the permeability of vessels causing the release of fibrinogen which triggers coagulation. Coagulation and fibrinolysis are regulated by tissue plasminogen activator (tPA) and urokinase-type plasminogen activator (uPA), respectively, with plasminogen activator inhibitor-1 inhibiting both and favoring the EC detachment from the matrix (16). Clots are dissolved by plasmin which also activates metalloproteinases (MMP), the binding of MMPs to the protein Vitronectin (facilitates binding to αvβ3) stabilizes it. The secreted MMPs encourage the breakdown of the basal lamina by the EC and then its matrix. While collagen type 1 is being degraded by the MMPs, the RGD (Arginine–Glycine–Aspartate) sequences are exposed enhancing its binding to αvβ3 integrin. The formation of a new basal lamina which blocks the contact between the EC and collagen type 1 stops the progression. bFGF and VEGF/protein kinase C induce the integrins αvβ3 and αvβ5, respectively (16).

After the formation of these vessels, there is the need to determine their arterial-venous identity. Notch ligand and receptors are regulators of cell fate, while regulators of arterial fate include VEGF and Ephrin B2 with Ephrin B4 and Coup-TFII being venous cell fate regulators (17).

**Figure 2:**
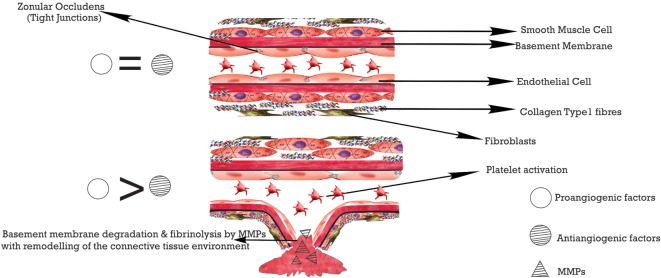
**ECM modification with the degradation of the basement membrane during angiogenesis**.

Overall, a couple of factors help to halt the angiogenic process, and these include tissue inhibitors of MMPs (TIMP), cytokines such as IL-4, IL-10, IL-12, thrombospondins-1 and 2, MMP2 c-terminal lytic fragments. Other angiostatic agents include endostatin, angiostatin, maspin, and TGFβ (16). See Figure 2.

**Figure 3:**
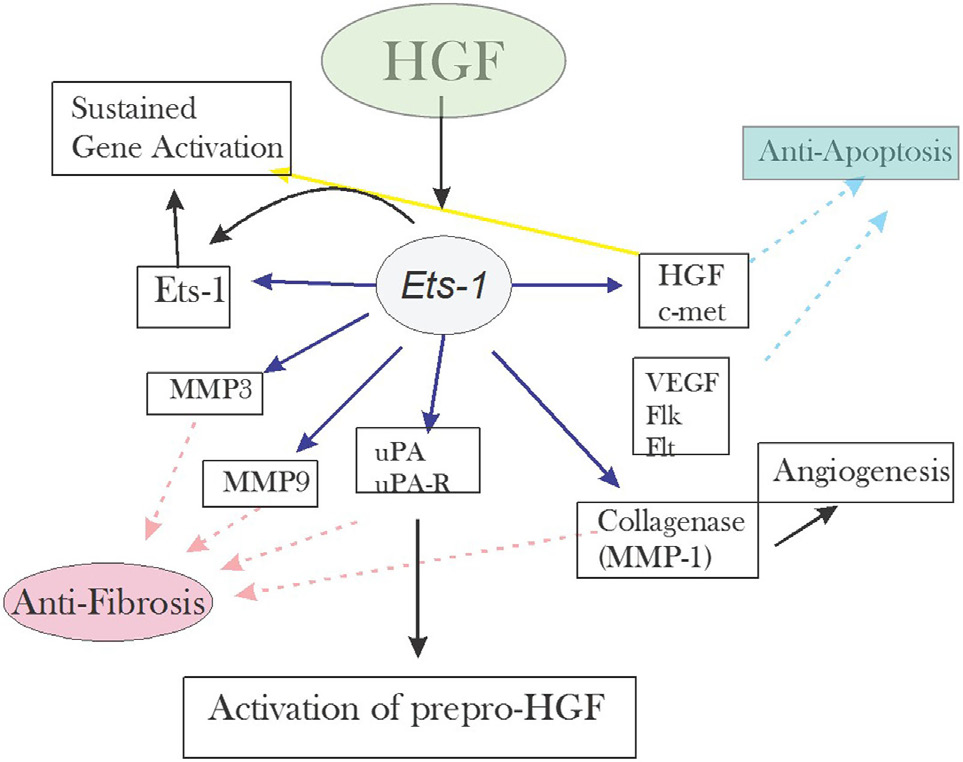
**Redrawn from Ref. (19, 20)**.

Hepatocyte growth factor considered the angiogenic factor with the most potency affects both the ECs and SMCs. The binding of HGF to its C-met receptor activates Ets-1, which then facilitates the expression of the factors necessary for angiogenesis and regeneration (18). Figure 3 below summarizes the event.

However, during ischemic/hypoxic states, the expression of hypoxia-inducible factor (HIF)-1 is induced. HIF is a transcription factor which encodes for the angiogenic cytokines. It has two subunits: the alpha is regulated by oxygen while the beta is constitutively expressed (21). These angiogenic cytokines with their respective receptors include VEGF/VEGFR1, placental growth factor/VEGFR2, stromal-derived factor (SDF)-1/chemokine receptor 4 (CXCR4) (22). In muscle ischemia, for instance, there is an elaboration of a higher than normal SDF-1, which facilitates the mobilization of hematopoietic stem cells (HSCs) from the bone marrow niche to the peripheral circulation. The stem cells are recruited to the site of ischemia where the ligands SDF-1, stem cell factor, and fibronectin are bound by their respective receptors CXCR4, C-Kit, and α-4 integrin on the stem cells.

In tissue hypoxia, the elaborate intracellular oxygen sensor proteins, the protein hydroxylase domain-containing proteins 1-3, and the HIF-1 and 2 analyze cellular oxygen content and induce a response to counteract the deficient oxygen supply (23). The abundance of HIF is not solely controlled by PHDs but also by nitric oxide (NO) even in normoxic conditions. NO influences the accumulation of HIF-1a protein by preventing its degradation independent of gene transcription and translation (24). VEGF is induced in an HIF-1a dependent manner during EC hypoxia and HIF-1a and 2a cannot be substituted for one another as they perform distinct roles in angiogenesis (23).

Nitric oxide, similar to VEGF, is a potent angiogenic and vascular permeability factor which plays a vital function in both physiological and pathological angiogenesis (25). Three isoforms of NO synthase exists, which are neuronal NOS, inducible NOS (iNOS), and endothelial NOS (eNOS). VEGF enhances NO synthesis and likewise induces the expression of iNOS and eNOS (expressed constitutively in vascular endothelial cells) (23). Thus the assumption that vascular EC eNOS synthesizes NO in response to VEGF, and this NO mediates the increased vascular permeability and angiogenesis induced by VEGF. NO synthesized by iNOS also mediates both vascular permeability and angiogenesis, however to a lesser degree than eNOS (23). The NO enhanced angiogenesis may be more of pathological such as in solid tumors than the physiological angiogenesis which occurs in development and vasculogenesis.

Other angiogenesis enhancing factors (mobilizers) include erythropoietin, granulocyte colony stimulating factor, statins, estrogen, exercise, and peroxisome proliferator-activated receptor (PPARγ), and these utilize ECs or endothelial progenitor cells (EPC) from circulation, bone marrow, or exogenously administered.

This molecular background drives the diverse studies and therapeutic approaches used in the field of stem cells and regeneration, and this knowledge is fundamental and is key to the understanding of this review.

## Stem Cells in Ischemia

Several treatment lines are available for patients with CVDs especially those who might have suffered an MI. These ranges from aspirin to nitroglycerin, B-blockers, and anticoagulants. However, those with occlusive symptoms are subjects for immediate reperfusion therapies with thrombolytics/percutaneous coronary intervention, commonly called coronary angioplasty. Coronary bypass graft surgery (CABG) is usually done when all reperfusion therapies have failed. Though these procedures undeniably have saved a lot of lives, the quality of life somewhat is still not optimal, especially in the case of an MI with a resulting permanent damage (Scar). On the other hand, for limb ischemias, drug-eluting stents/drug-coated balloons in femoral and popliteal arterial segments, others include adventitial anti-inflammatory therapy, stimulation of the spinal cord, and intermittent air(pneumatic) compression.

Also, several clinical trials using angiogenic factors like VEGF, FGF, HGF have bene tried on patients with not so encouraging results (26). Thus, the enormous clinical burden posed by CVDs have immensely stimulated directions toward gene and cellular therapies (27). Stem cells or progenitor cells have been proven to have the potentials to differentiate into any tissue type in the body including the cardiovascular system (such as endothelial cells, smooth muscles, and CMCs). With this potential, we see stem cells not just being able to repair or regenerate tissues but also facilitate revascularization.

### Stem Cells Utilized in Ischemic Models

Several studies have been carried out in animals using diverse populations of (stem) cells for the treatment of CVDs and hind limb ischemias. These includes embryonic stem cells (ESC) (28, 29), mesenchymal stem cells (MSC) (30, 31), bone marrow mononuclear cells (BMMNC) (32, 33), endothelial progenitor cells (EPC) (34, 35), mature ECs (34), marrow-derived stromal cells (36), peritoneal macrophages (37), polarized macrophages (38), adipose tissue-derived stem cells (39), autologous cultured adipose-derived stem cells (40), saphenous vein progenitors (SVPs) (41), pericytes (42).

Despite the benefits of these stem cells, tumor formation and immune intolerance have been a major drawback to their use. Among others, MSCs are considered one of the main and the most attractive sources for stem cell therapy for reasons such as low tumorigenic potentials (43, 44), ease of access as well as minimal immunogenicity (45). Also, ESCs are quite immune privileged but they may trigger some inflammation *via* their derivatives (46).

Overall, most researchers believe that the benefits derived from stem cells do not occur through the transdifferentiation of stem cells but rather from their paracrine functions which include the release of cytokines and growth factors (47–49).

## Stem Cells Delivery, Results, and Discussion

Stem cells have been transplanted or administered in the setting of ischemia through various routes. In the case of an MI from the clinical point of view, these have been through routes such as (i) transvenous infusion, (ii) intracoronary arterial infusion, (iii) direct intramyocardial injection with CABG, (iv) trans-endocardial injection using catheter, and (v) mobilization of stem cells. Zhou and colleagues documented the routes for the administration of stem cells in critical limb ischemia, these are the intra-arterial, intramuscular, or the combination of both. For an MI, the two most utilized methods are CABG with intramyocardial injection and the intracoronary infusion, while the intramuscular injection is the most preferred method during limb ischemia (50).

The transvenous route, though an easy approach for stem cell delivery in myocardial injury, relies majorly on a successful homing process and stem cell retention (51).

The intracoronary route of delivery encourages the homogenous administration of a maximum number of cells to the damaged site. However, the non-perfused areas do not benefit (52).

Direct intramyocardial injection is the most preferred method of delivery, particularly for patients with chronic heart failure (53). However, necrosed tissues are oxygen and nutrient deprived, and injected cells would not have healthy cardiac cells to provide paracrine support, hence a reduction in differentiation and graft survival (50).

The Table 1 below shows the advantages and limitations of the methods of delivery stem cells.

**Table 1:** **Advantages and limitations of routes of delivery**.

Delivery method	Advantages	Limitations
Intracoronary artery infusion	Direct infusion infarct-related coronary artery cell engraftment homogenously at infarct borders	Not applicable to occluded artery. Possible micro embolism during infusion
Surgical intramyocardial injection	Smaller number of cells required direct inspection	Increased risk of morbidity and mortality. Potential induction of arrhythmia. Need for a surgical approach
Transendocardial injection	Cell delivery in occluded areas	The risk of myocardial perforation. Cardiac tamponade and ventricular arrhythmia
Transvenous infusion	Non-invasive Simple delivery	Possible micro embolism Low cellular migration and differentiation. Homing to non-cardiac organs

*Adapted from Ref. (50)*.

Researchers have investigated best possible methods for stem cells delivery which will result in a successful neovascularization. The goal is ensuring a successful homing of the cells to regions of ischemia, prolonging survival, and retention of these cells, as less than 10% of cells are retained after 24 h and only about 1% after 4 weeks (54). Furthermore, methods that non-invasive are being considered because of the hazards associated with operative procedures. Taking stroke as a case study, injecting neural stem cells or mesenchymal cells directly into the lesioned brain might yield a remarkable result, but the use of a non-invasive technique could be used to avoid the associated operative hazards.

The new methods which have been used for the delivery of stem cells include

(a) Scaffolds
(b) Retrograde delivery
(c) Improved combinatorial approaches
(d) Stem cell priming or pretreatment
(e) Preconditioning
(f) Stem cell Exosomes
(g) Magnetic enhancement techniques
(h) Ultrasound (US) techniques using (a) microbubble destruction and (b) acoustic radiation force
(i) Enhanced homing technique
(j) Stem cell modulation techniques
(k) Mannitol-enhanced delivery

### Scaffolds in Stem Cell Delivery

Scaffolds are biological materials, which act as a template for tissue regeneration and thus guide the growth and formation of the new tissue. The use of scaffolds has been extremely useful in regenerative medicine in that it permits the combination of regenerative cells and angiogenic growth factors as well as allowing control of the cell microenvironment as well increasing cell survival and retention within the ischemic host milieu.

#### Matrigel

The encapsulation of cells enhances the formation of multicellular aggregates and also prevents *in vivo* migration of cells (55). hESC-ECs encapsulated in Matrigel (enMA-hESC-ECs) have been demonstrated to be superior to hESC-ECs alone in the treatment of limb ischemia as it permits use without direct incorporation enhancing sustained release of various growth factors like VEGF, GM-CSF, IL-6, and IL-8 thus exerting its functional potential through a paracrine effect (29). The encapsulation could also help to overcome the major side effects of transplantation such as teratoma formation and tumors (55).

#### Cardiogel

Cardiogel is a biodegradable 3-D cardiac fibroblast-derived extracellular nano-matrix scaffold (56) with a lot of therapeutic potentials for cardiac tissue ischemia. Cardiogel has been shown to support adhesion, differentiation, and proliferation of stem cells (BMSCs) as well as providing increased protection against oxidative stress when compared to Matrigel (57). Cells have been shown to adhere firmly and resist dislodgement even with trypsinization hence preventing flow off to redundant regions of the delivered cells (57).

#### Hydrogels

This biomaterial is very attractive as a scaffold because of its similarity to extracellular matrix and under rather mild conditions can be processed. Its delivery is minimally invasive, and its degradation can be designed in a timely way to coincide with the process of angiogenesis (58). Different hydrogel matrices, either natural or synthetic, have been employed as carriers for delivery of cells or growth factors. Naturally occurring molecules that can be utilized include, collagen, fibrin, gelatin, and hyaluronan, alginate, chitosan which are polysaccharides. The synthetic hydrogels include poly (lactic-co-glycolic acid) (PLGA) and polyethylene glycol (PEG).

##### Collagen

Less definite matrices, like collagen, were used in the past to provide support for locally injected cells with the restoration of vascular networks (59). These matrices, however, did not provide the needed controlled release of specific growth factors for clinical applications.

However, this downside has seen improvement over the years. For instance, Matsuse et al. (60) designed a combinatorial delivery system using collagen sponge as a matrix for the transplanted neural stem cell (61). Also, it has been combined with gelatin microspheres to enhance the period of vascularization by the gradual release of bFGF. Likewise, to strengthen the use of collagen materials in regeneration, Shi et al. (62) combined three different types of collagen materials in the regeneration of cardiac tissue (demineralized bone matrix collagen scaffolds, collagen gels, and collagen membranes).

##### Fibrin

The traditional way of using fibrin for delivery is a physical mixture of the factors with thrombin and fibrinogen during the process of coagulation (63), however, using this method results in an uncontrolled release kinetic of the angiogenic factors VEGF and bFGF, usually within 24 h (64). New approaches developed to overcome this include covalently linking fibrin to growth factor by factor XIIIa transglutaminase activity during coagulation (65). Another is the use of the engineered variant of VEGF121 (α2-PII-8-VEGF121), which attaches itself to fibrin during the coagulation process and this prolongs VEGF expression and retention within the fibrin matrix (65). Jeon et al. in their mouse limb ischemic model study, BMMNC and bFGF were transplanted using fibrin as a scaffold. Fibrin was shown to increase density and survival of transplanted cells (32) as the attachment of cells to matrix enhances survival (66, 67). Despite the benefits, fibrin has its drawback which is its structural weakness with regards to withstanding the dynamic nature of the *in vivo* physiological environment, hence the need for the introduction of synthetic biomaterials (PEG, PLGA).

##### PEG and PLGA

PEGylated hydrogels are designed to be biodegradable in response to tissue proteases. The combination of MSCs and ECs with PEG hydrogels resulted in the formation of extensive tubule-like structures and the differentiation of MSCs further enhanced its stability into SMC lineage (68). PEGylated fibrin hydrogels were used in a mouse model of MI to deliver MSCs because of the drawback of fibrin (69), and this demonstrated a 15-fold cell retention. Also, there was a substantial reduction of apoptosis within and around the scar region and the resulting vascularization led to a significant improvement in the contractile function of the heart.

Poly (lactic-co-glycolic acid) hydrogels also have been used to allow a controlled release of angiogenic factors like VEGF and pre-encapsulated PDGF (70) but without protection in the tissue environment. The PLGA microparticles, an injectable scaffold was designed for the benefit of a prolonged release over months and the choice of polymers with varying release profiles spanning from 1 week to a couple of months controls its gradual degradation into non-toxic products (71). Saif et al., in their study, compared a dual (HGF and VEGF) to a triple (HGF, VEGF, and Ang-1) combination of pro-angiogenic factors in a mouse model of hind limb ischemia. This study was able to demonstrate how Ang-1 helps in the stabilization of newly formed vessels over VEGF. Thus, more vessel formation was induced with the triple combination when compared to the dual therapy (71).

##### Hyaluronic Acid (HA) and Heparin

Hyaluronic acid, a glycosaminoglycan with its angiogenic effect dependent on its molecular weights. The high molecular HAs inhibit angiogenesis, while the low HAs enhanced the proliferation and migration of endothelial cells (72, 73). HA can be chemically modified to create a hydrogel that is biocompatible with macropores that retain and release angiogenic growth factors *in vivo* (74, 75). On the other hand, heparin incorporation into hydrogel helps to sequester growth factors thereby slowing down their release while still retaining their bioactivity. Heparin stabilizes the growth factors preventing its degradation by ECM proteinases (76, 77).

Pike et al. designed a hydrogel of HA and gelatin conjugated with growth factors VEGF and bFGF and demonstrated that incorporation of heparin resulted in an extended release of the growth factors, while the *in vivo* bioactivity of the growth factors was maintained (78).

##### StarPEG-Heparin

StarPEG-heparin is a biohybrid hydrogel which is composed of a star-shaped (multi-armed) poly (ethylene glycol) which is the “starPEG” coupled with heparin (79). This hydrogel can serve as a reservoir and an adaptable release system for heparin-binding factors (VEGF and FGF-2); thus, it is an ideal material for a multifactorial delivery system (80). Likewise, this hydrogel was shown to be suitable for the culture of human umbilical vein endothelial cells, and by adjusting the physiochemical structure of the hydrogel independent of the biomolecular functionalization, the EC behavior such as morphology, proliferation, and survival could be modified (80). Likewise, the attractant for EPCs, the angiogenic protein SDF 1 alpha (SDF-1α) have functionalized StarPEG-heparin, and by the incorporation of varying concentrations of MMPs, controlled cleavage, and release of varying amounts of SDF-1α can be achieved (81).

##### Alginate

These type of hydrogels have been used to encapsulate angiogenic growth factors VEGF and bFGF (82), hence comparing their angiogenic capacities (83). Neovascularization has been promoted using alginate hydrogels by the localized and sustained delivery of VEGF and bFGF, but a low dose angiogenic signal delivery is essential for the production of a functional vasculature because unregulated hemangioma formation may be a side effect of VEGF and bFGF overdose or vascular leakage that results in edema (58).

Heparinized alginate has been introduced to overcome the initial burst release of growth factors, and the ensuing sustained release of bFGF, for instance, has been shown to treat porcine with MI (84) effectively. More recently, alginate-sulfate-hydrogels (a modified alginate hydrogel) was conjugated to HGF in a rabbit model of hind limb ischemia, which resulted in considerable neovascularization (85).

Further modifications include the RGD (Arg–Gly–Asp) modified alginate gel, which has been shown to improve the attachment and growth of MSC, also facilitated angiogenic growth factor expression. MSCs encapsulated in RGD modified alginate gels promoted repair of the heart muscle with angiogenesis by improved cell survival in an ischemic environment (86).

##### Poly-l-Lactic Acid (PLLA)

Poly-l-lactic acid has equally been coupled to RGD to form RGD-g-PLLA synthetic scaffold. These three have been shown to be good for EPC-targeted delivery *in vivo* as it was proven to promote vessel regeneration in mouse models for dermal wound (87). However, this scaffold was limited by the fact that it appeared to degrade rather slowly. As established, the success of transplanted cells is largely dependent on the adequate and timely degradation of the scaffold which is necessary for the migration of the delivered stem cells (87).

#### Polyvinylidene Fluoride-Tetrafluoroethylene (PVDF-TrFE) Scaffolds

The piezoelectric scaffold, electrospun PVDF, and PVDF-TrFE have recently been produced for tissue engineering purposes. This scaffold has proven to be attractive for cardiovascular tissue engineering since the heart is an electroactive tissue (88).

The PVDF-TrFE scaffolds as studied by Pamela et al. revealed that they supported the attachment of and survival of embryonic stem cell CMCs (mES-CM) and endothelial cells (mES-EC) and they both expressed classical endothelial markers such as PECAM1, eNOS, and TM on the scaffolds. The scaffold also supported the contractile action of mES-CM, while it displayed a higher expression of Notch1 an arterial marker suggesting that it may support arterial specialization. Also, the mES-EC-PVDF-TrFE scaffold retained their ability to uptake LDL, a known feature of mature endothelial cells (88).

However, further studies need to be done to determine the long-term benefits of *in vitro* cultures using PVDF-TrFE scaffolds, detailed characterization of its piezoelectric activity, as well as its functional integration *in vivo* (88).

### Retrograde Delivery

In the setting of an MI, blood flow to the distal segment will be cut off and as such infused stem cells will not be able to get to the region of ischemia. In this regard, Wu et al. studied the retrograde delivery of cells *via* the coronary venous vessel which was initially described by Baklanov et al. This procedure helps to circumvent the blockage to deliver stem cells to the area of ischemia. Unlike the delivery of cells into a coronary arterial vessel which might trigger an ischemic event, Wu and colleagues demonstrated that the temporary blockage of the coronary sinus did not result in any hemodynamic change and the procedure was well tolerated in the setting of a myocardial injury (50). The advantage of this method is in its ability to administer a lot of cells, low cost, and less invasive when compared to other delivery methods (50).

### Improved Combinatorial Approach

Though the EPCs have a critical role in the formation of blood vessels, the functions of other cells and angiogenic factors cannot be undermined hence the thought of possible combinations. Previous studies have utilized a lot of combinations, but a more recent and classic one is the study by Odent and colleagues, 2015 (89), where they combined the two types of EPC populations. Two distinct types of EPC exist and are described in accordance to how they appear in culture (90, 91). These are (1) early outgrowth (EO-EPC), also known as the circulating angiogenic cells (CAC) which appear about 7 days in culture. The proliferative potentials of EO-EPC have been observed to be limited, but with increased secretory activities (89). (2) The late outgrowth (LO-EPC), also known as the endothelial colony-forming cells (ECFC) are circulating EPCs, which are rare and only develop after 2–3 weeks on culture. Both of these cells share common EC markers (CD31/PECAM, KDR/VEGFR2/Flk-1, CD144/VE-Cadherin) and monocyte markers (CD14, CD45) (92). Concurrent delivery of ECFC and CAC secreting soluble factors has been proven to be of immense benefit for the hind limb ischemia (89). The delivery (i) increases the storage of the cells delivered to the area of ischemia, (ii) pulls in mural cells to facilitate the stability of the new vessels, and (iii) enhances neovascularization and regeneration of muscle tissue. The blood from the umbilical cord has been shown to possess a higher number of ECFC with a significantly greater proliferative potential when compared to cells obtained from the adult peripheral circulation (93), hence the justification for the use of umbilical cord blood for this study. This combination demonstrated a better vascular regeneration, and this was thought to be due to the first endothelial sprouting followed by pericytes ± SMC recruitment. CAC contributed immensely to vessel maturation by the secretion of angiogenic and vasculogenic factors that promoted survival and proliferation of delivered cells and organized the intercellular junctions between the SMC and ECFC. These factors include VEGF, SDFa (secreted in very high amounts in both normoxic and hypoxic states), PDGF, Ang2, IL-8, ET-1, MCP-1, 10.

Also, Avolio et al. also recently combined human pericytes SVPs (boosts vascularization) with cardiac stem cells (CSC) (promotes cardiomyogenesis) in a mouse MI model (94). This combination additively resulted in a scar size reduction with increased collateralization. The study showed that both cells contributed distinctly rather than cooperatively. SVPs, when compared to CSCs, were shown to have a better incorporation rate and notably, both cell populations were located far from one another *in vivo*. Particularly, SVPs individually secreted larger amounts of angiogenic factors to include microRNAs (miRs)-132. However, coculture suggested some inhibitory effects where these values were significantly reduced in both populations, whereas SDF-1α was significantly elevated in the coculture (94). Though tissue engineering might be able to harness benefits from both cell populations; this, however, has to be carefully tailored to consider both the existing competitive and cooperative interactions.

Previous combinations include that of EPCs with smooth muscle progenitor cells used in a mice hindlimb ischemia model (95). Here, the combination therapy examined the angiogenic roles and benefits of Ang-1/Tie2 signaling.

Another recent study combined EPCs with MSCs (a source of angiogenic factors and pericyte progenitors) (96). This combination suggests that bone marrow can potentially generate a vast array of chemokines, growth factors, and extracellular matrix molecules derived MSCs (97) with molecular and cellular properties closely similar to that of pericytes (98, 99).

### Stem Cells Priming/Pretreatment

#### Stem Cell Senescence

The senescence of stem cells result in the inability of the cells to regenerate injured or ischemic tissues and is thus very detrimental for the life of the organism. The natural aging process and pathologies like diabetes mellitus (DM) contribute to cellular senescence. Cellular senescence has been a challenge for therapeutic tissue regeneration, and a lot of studies have demonstrated that statins, estrogen, high-density lipoprotein, and Insulin-like growth factor-1 can trigger the activity of telomerase (100–103). Also, hypoxia has been shown to inhibit EPC senescence by activating TWIST (104). The priming of stem cells before transplantation is a newly emerging technique in overcoming senescence in stem cells.

When stem cells become senescent, they undergo a couple of changes that include (i) loss of expression of functional markers for ECFC such as, CD34, CXCR4 (which is SDF-1a receptor and though to delay senescence of subpopulations of EPC), VEGFR2, and c-Kit (linked to the recruitment, mobilization and survival of EPCs) (105) and (ii) they express low levels of SMP30 protein, which is a marker of aging and senescence. SMP30 prevent apoptosis in the cells by the inhibition of the caspase cascade.

Fucoidan, the sulfated polysaccharide, is a marine product extracted majorly from diverse species of brown seaweed and brown algae (106). Some functions of Fucoidan have previously been itemized such as in combination with FGF-2 have been shown to enhance the angiogenic activities of EPCs (107), increase migration of endothelial cells induced by VEGF165 (108). Lee et al. went further to show the effect of Fucoidan on EPC senescence. Which include (i) restoration of the expression of ECFC functional markers as described above, and this has been shown to be dose-dependent; (ii) prevention of the senescence of ECFC *via* the phosphorylation of Akt, also with increased and decreased SMP30 protein and p21 levels, respectively. Akt phosphorylation is *via* the integrin-FAKAkt signaling pathway. Focal adhesion kinase (FAK) promotes survival of cells *via* PI3K/Akt pathway (109, 110). This pathway indirectly modulates the expression of the cell cycle regulators cyclin-dependent kinases (Cdk2 and Cdk4). (iii) Acceleration of senescent ECFC’s proliferation *via* phosphorylation of FAK-ERK, ERK signaling regulates various cellular functions, mainly survival and proliferation (111). ERK signaling indirectly controls the expression of p21 a Cdk inhibitor (cell cycle repressor).

Diabetes can induce a dysfunctional EPC, and microarray studies have confirmed this in a homogenous population of type 1 DM patients (112). Several studies have shown that transplantation of autologous EPCs in the setting of DM foot or the utilization of EPCs from patients with DM yielded very poor neovascularization. Diabetes results in a significant downregulation of a crucial pro-angiogenic glycoprotein Osteopontin (OPN). Vaughan et al. demonstrated that pre-exposure of autologous EPCs to OPN might enhance their efficacy before transplantation. Thus, dysfunctional EPCs can be induced to secrete angiogenic proteins when acted upon in an autocrine manner by OPN (112).

Also, Avolio et al. treated senescent CSC gotten from a decompensated heart with rapamycin and resveratrol and these cells induced cardiac repair when injected into an infarcted mouse heart with the reduction in CMC senescence by reducing the secretion of IL1β (113).

#### miRNA Pretreated Stem Cells

microRNAs, a large family of small non-coding RNAs (22–24 nucleotides long), they endogenously regulate gene expression directly or indirectly and play a vital role in cell proliferation, differentiation, and apoptosis (114). The transfection of MSCs with specific miRNAs enhanced their survival and their potential for lineage differentiation thus potentiating MSCs reparative function in ischemia.

Mesenchymal stem cells have been transfected with several miRNAs such as miR-378 (115), MSC Anti-377 (116), miR-126 (117). MSCs transfected with mi-R378 compared to the control, showed a more rapid proliferation, decreased apoptosis, decreased BCL2 levels, upregulated BAX, reduced expression of TNF-α, decreased TUSC-2, and formed a higher number of vascular branches. Mi-378 transfection also augmented the expression of VEGF-α, PDGF-β, and TGF-β1 (115). However, an *in vivo* study of MSC-miR-378 affection is yet to be conducted.

miR-377 binds directly to VEGF and negatively regulates its expression (116). Wen et al demonstrated a significant improvement in myocardial angiogenesis when treated with MSCAnti-377, also the degree of fibrosis was observed to be less when compared to MSC-null injected cardiac tissue (116).

Huang et al. demonstrated that MSCs pretreated with miR-126 encoded in a lentiviral vector survived for a longer period of time under hypoxic environment (117). MSC-miR-126 efficiently expressed miR-126 for a minimum of 2 weeks at the injected site with no demonstrable adverse effects on cell viability. The overexpression of miR-126 led to the upregulation of VEGF, Notch ligand DII-4, and bFGF in the MSCs. It also improved the survival of MSCs and increased functional angiogenesis in the ischemic heart tissue. However, the study could not explain how the overexpression of miR-126 enhanced the paracrine effects of MSCs.

### Preconditioning

This approach includes (i) electrical stimulation and (ii) ischemic preconditioning both of which has been proven to promote post-transplantation stem cell survival and improved heart function by probably altering exosome contents and functions. It has been proposed that the beneficial effects of either approach occur from induction of cellular stress (118).

Kim et al. showed that CSCs preconditioned with electrical stimulation demonstrated a decreased apoptosis both *in vitro* and *in vivo* by upregulating the pro-survival pathways PI3K/AKT and FAK causing the release of connective tissue growth factor (CTGF) (119). miR-378 was shown to regulate the expression of CTGF. CTGF has known effects on tissue repair, fibrosis, scarring, protection against reperfusion injury, ES adhesion and survival. Also, CTGF has been shown to bind to a number of growth factors and mediators that direct a variety of cellular pathways (119). However, their study did not explore the impact of electrical stimulation on exosomes as well as the cytoprotective effects of electrical stimulation.

Ischemic preconditioning is a process where cells are exposed to repeated cycles of anoxia with intermittent re-oxygenation. Kim and colleagues, in a study of ischemic preconditioning, demonstrated an intracellular increase in the levels of miR-210. The expression of this miRNA resulted in the activation of the AKT/ERK1/2 survivals pathways targeting the Caspase-8-assoicated protein 2, an initiator of apoptosis (119). A recent study by Feng et al., not only demonstrated the expression and loading of the antiapoptotic miR-22 in exosomes but was able to capture the dynamic intracellular course of exosomes and the extracellular shedding of miRNA-loaded exosomes. miRNA-22 reduced apoptosis in ischemic CMCs by directly targeting methyl binding protein 2, ameliorated fibrosis, and enhanced cardiac function (120).

The window of protection offered by electrical stimulation is shorter when compared to ischemic preconditioning, and this further supports their different molecular signaling pathways (117).

### Stem Cell Exosomes

Stem cells demonstrably secrete paracrine factors not only in naked forms but also membrane bound vesicles such as exosomes among others (121). Exosomes are secreted vesicles, 30–100 nm in diameter and are also referred to as extracellular vesicles. They are essentially composed of a host of biologically active molecules, such as messenger RNAs (mRNAs), miRs, and proteins (122). Exosome formation is initiated from the invagination of the endosomal membrane that results in the formation of intraluminal vesicles or multivesicular bodies (MVBs), which in the process accumulate cytoplasmic molecules such as mRNAs, miRs, and proteins (123). The ILVs/MVBs fuse with the plasma membrane and are released into the extracellular environment by exocytosis as exosomes. MVB formation has been reported to be mediated by the endosomal sorting complexes required for transport (ESCRT) and other ESCRT independent machinery (124). The other molecules involved in the exosome formation are the tetraspanins (CD9, CD63, and CD81) which are expressed on exosomes and can be used as exosome identity markers (121).

Exosomes isolated from different types of stem cells such as MSCs, iPSCs, cardiac progenitor cells, ESCs, and CD34+ have been utilized in the study of cardiac and limb ischemia. In these studies, exosomes were shown to significantly suppress apoptosis (120, 125–127), stimulate angiogenesis (125, 127–131), reduce infarct size (128, 132, 133), and recover cardiac function (125, 127, 128, 131–133). Also, they have been shown to be enriched in miRs such as miR-22 (120), miR-19a (133), miR-133b (134), miR-146a (125), miR-294 (131), and sonic hedgehog, a pro-angiogenic factor (135).

Despite the demonstrable angiogenic benefits of exosomes for CMC regeneration after an acute MI, MSC-derived exosomes have also been shown to promote tumorigenesis *in vivo* by stimulating VEGF in tumor cells (136). Therefore, a technique to specifically deliver the exosomes to target tissues needs to be explored.

### Magnetically Targeted Delivery of Stem Cells

This study involves the use of biocompatible nanoparticles that are magnetically responsive for the targeted transplantation of stem cells so as to facilitate their retention in the region of therapeutic interest. Furthermore, these nanoparticles can be utilized for tracking and monitoring of cells *in vivo* with the use of magnetic resonance imaging.

#### Magnetic Nanoparticles

The superparamagnetic iron oxide nanoparticles are typically made up of a core of magnetite or maghemite which are both naturally ferromagnets, but at specific radii, they become superparamagnets (137). Superparamagnet iron oxide NPs (SPIONs) can broadly be categorized by their size, which is relevant to the field of study as well as their application. These include the very small superparamagnetic iron oxide NPs, less than 10 nm; ultra-small superparamagnet iron oxide NPs, 10–50 nm; and lastly the SPIONs, 50–180 nm, which are employed in the field of stem cells (137).

#### Stem Cell Tagging with SPIONs

**Figure 4:**
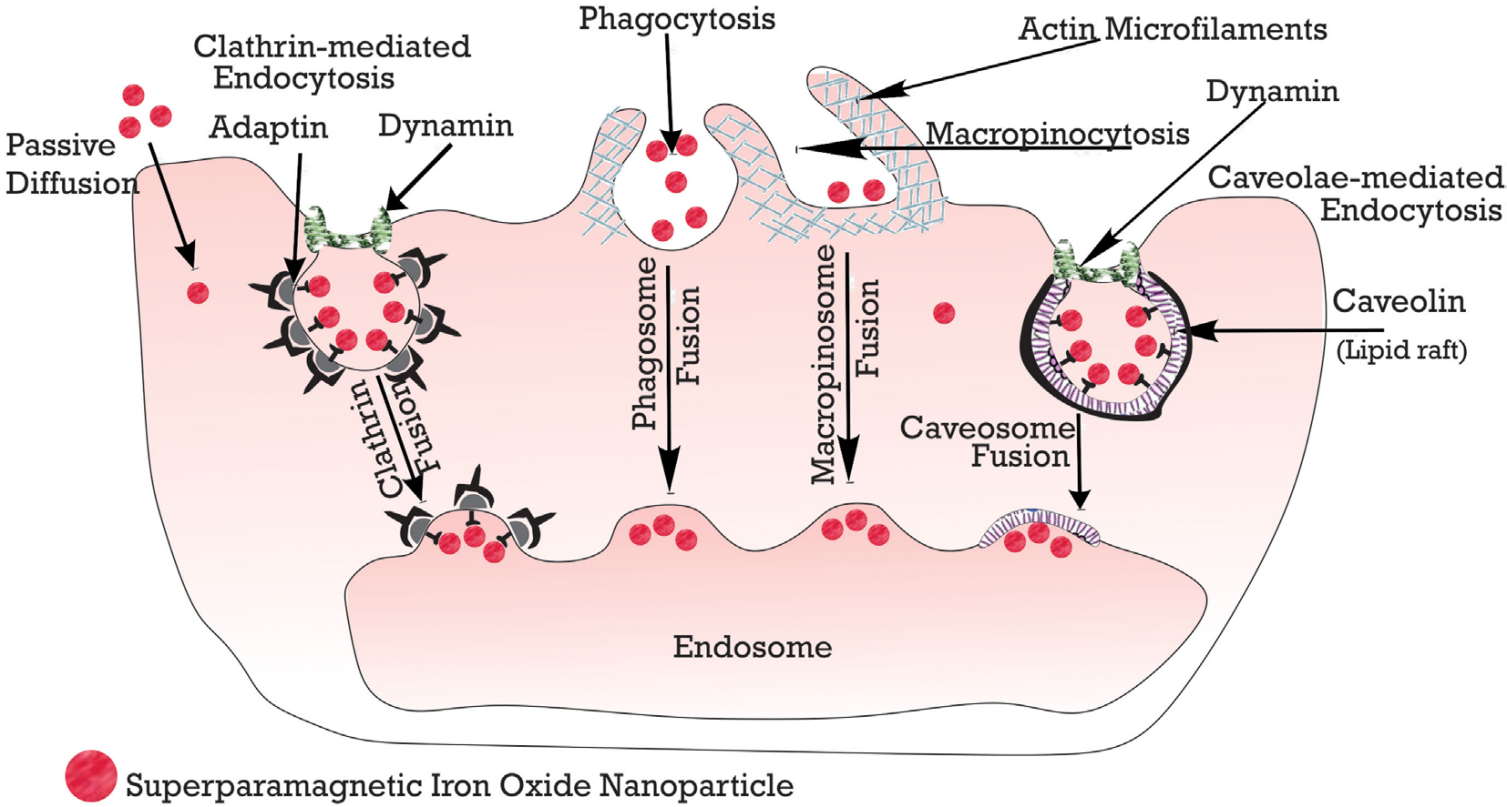
**Schematic overview of tagging stem cells with SPIONs**.

Stem cells are tagged with the nanoparticles using any of these methods: (i) passive diffusion, (ii) phagocytosis, and (iii) endocytosis, which can be macropinocytosis, clathrin, or caveolae-mediated endocytosis and other variants that are independent of clathrin or caveolae (138). However, mostly utilized is the endocytosis, in which the cells are incubated for about 12–48 h (139). Below, in Figure 4, is a schematic summary.

#### Delivery of Stem Cells Tagged with SPIONs

Several studies have utilized the SPION-tagged stem cells in vascular injury. Kyrtatos and colleagues used CD133+ EPCs, and they targeted these tagged cells using a magnetic device at the site of catheterization in the common carotid artery of a rat hoping to prevent re-obstruction post angioplasty. The engrafted CD133+ tagged cells to the area of injury had 5.4-factor increase about the control. The SPION technique has also been used in heart models to overcome the “Washout” or “displacement” of stem cells during cardiac contraction or coronary blood flow (140). Vandergriff and colleagues, in their study, used Feraheme, coupled with heparin (H) and protamine (P) sulfates forming complexes of FHP (139), and then incubated the cardiospheres with these complexes. The tagged cardiospheres experimented on a rat ischemic heart model with a magnetic field applied. Exposure to the magnet increased the rate of cell retention by three folds about the control. Also, there was no remarkable inflammatory difference (CD68 marker used) about control demonstrating its safety with improvement in cardiac morphology, remodeling, and scar attenuation.

Other areas to be considered in this delivery method include (i) the number of cells (dose), (ii) the magnetic intensities, (iii) duration of the application of the magnet, and (iv) cell size. Cheng, in his study in 2012 (141), in a rat ischemic heart model injected iron microsphere labeled cells derived from cardiospheres into the left anterior coronary artery and a 1.3 T magnet placed 1 cm above the heart. The 24-h cell retention was enhanced by a factor of 5.2–6.4 about control. They also demonstrated that the infusion of 1 × 10^5^, 3 × 10^5^, or 5 × 10^5^ cells resulted in no elevation of serum troponin levels, but higher cell number (1/2 × 10^6^) raised troponin levels with no improvement in cell retention. This effect was attributed to microvascular obstruction (141).

After that, Shen et al. compared the effect of different magnetic intensities for MSC targeting for myocardial ischemic repair. The left ventricle was injected with SPION-tagged 1 × 10^6^ MSCs with the application of magnetic intensities of 0.15, 0.3, and 0.6 T (142). This study showed a magnetic dose-dependent increase in cell retention, but at 0.6 T, there was no significant therapeutic outcome as MSC engraftment was confirmed to be low. Microembolism was linked to the therapeutic outcome observed at the too high magnetic strength (142). However, a different study affirmed that the cell dosage (1 × 10^6^) could also result in micro embolism regardless of magnetic intensity (141). So far, 10 min have been utilized for the magnet application, time. However, this is yet to be fully optimized (141) and also the smaller cells might less likely result in micro embolisms, so embryonic-like (2–4 μm) stem cells have to be looked into Ref. (142).

**Figure 5:**
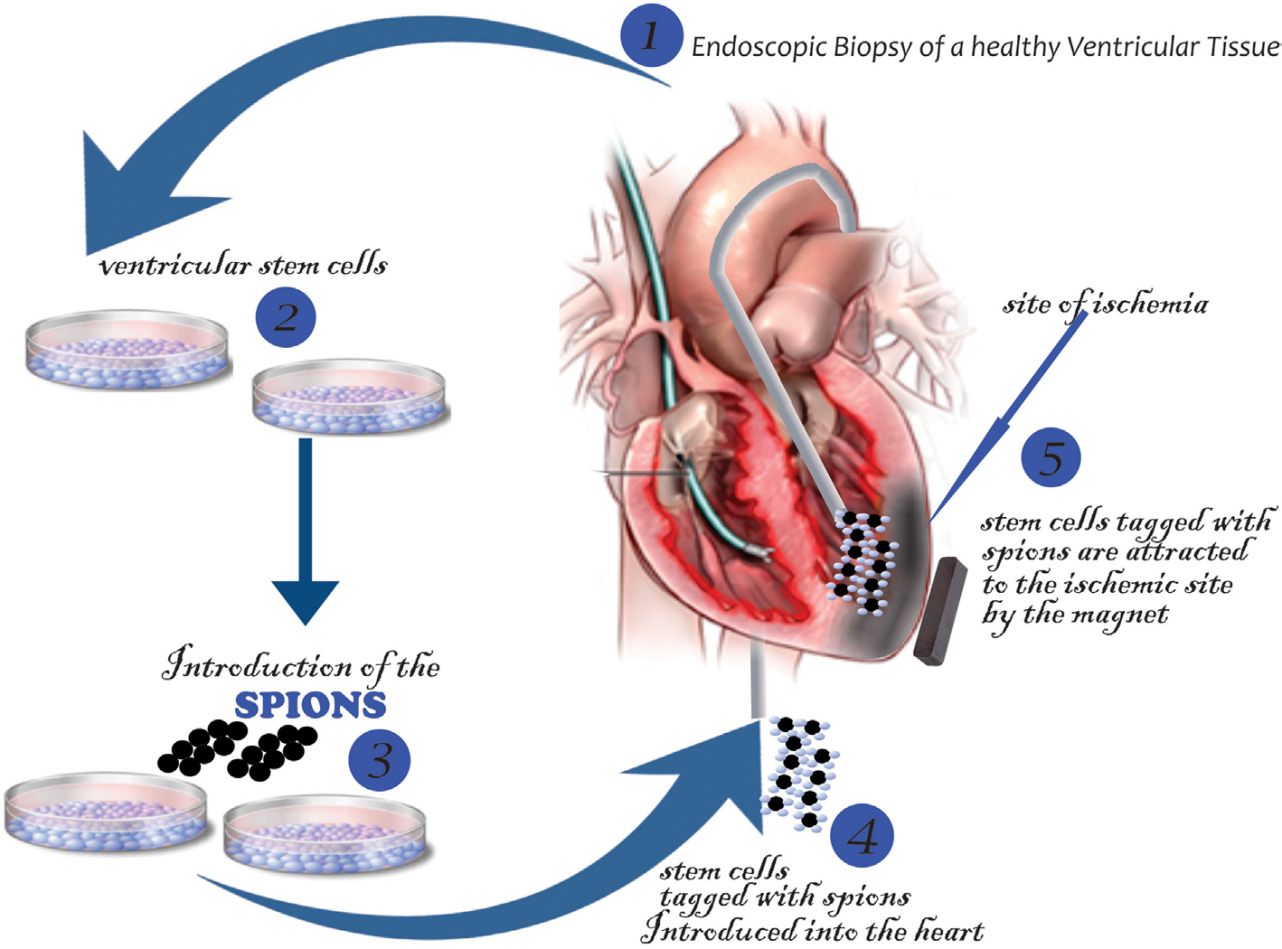
**Delivery of stem cells tagged with SPIONs**.

For its application in humans, Cheng and Vandergriff et al. proposed the schematic below in Figure 5.

### US in Stem Cell Delivery

**Figure 6:**
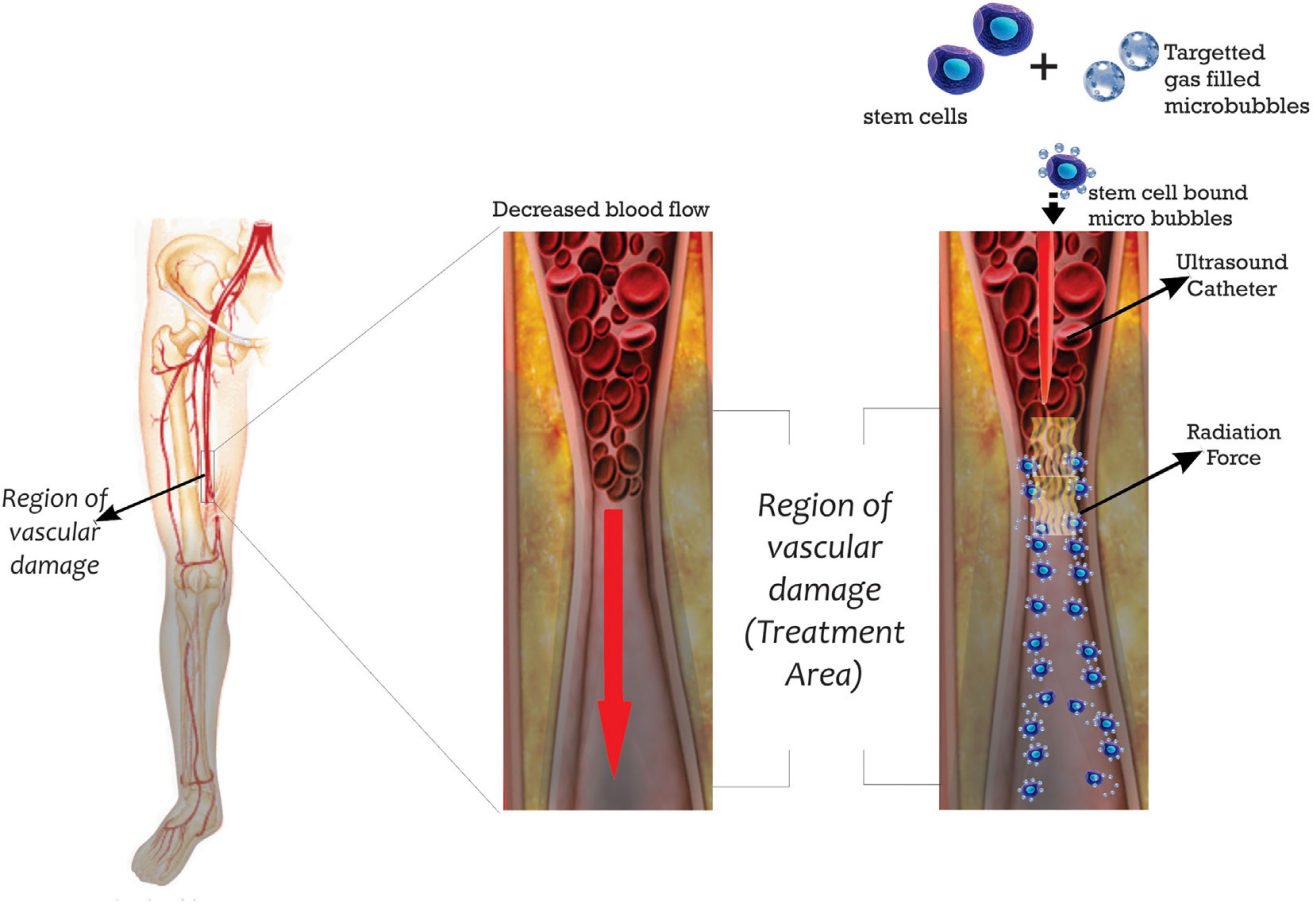
**US enhanced delivery of stem cells bound microbubbles**.

Ultrasound is another novel method of stem cell delivery where acoustic force is used to enhance delivery of stem cells to target sites. There are currently two approaches: (i) the US ability to displace cells toward the target site and (ii) the US directed modification of the target site microenvironment. The US delivery systems utilize microbubbles for its function. The first description of the delivery of stem cells using US radiation was by Toma et al. and was based on the intravascular catheter displacement of MSCs coated with gas filled, lipid-shelled microbubbles (143). The schema in Figure 6 demonstrates its possible design in humans.

The attachment of the microbubbles to the surface was catatonically enhanced, and microbubble number of about 7–11 facilitated the greatest adhesion of MSCs (143). However, increasing microbubble coating resulted in a corresponding decrease in adhesion of cells, and this effect was attributed to physical interference by the microbubbles (143). The target site MSC enrichment was outstanding being up to a factor of 150 when compared to the SPIONs loaded EPC which yielded only a target site enrichment of a factor of 6 over control (141). The extended exposure to the microbubbles, however, resulted in a 5% excess MSC death more than control. In all, the cell type and the catheter used for this delivery will still require further studies.

The other approach is the alteration of the microenvironment of the target site by “US-targeted microbubble destruction” (UTMD). Ling et al. used this system in dog model 1 week after the MI, where a 1 MHz transducer with a 2 cm diameter was used to irradiate the left ventricular wall after the femoral vein was injected with 2 ml of microbubbles for 10 min (144). The UTMD can enhance permeability and rupture of the microvasculature (145) and also promote angiogenesis by stimulating the secretion of endogenous angiogenic growth factors (146, 147). The irradiated site resulted in some modification of the microenvironment, which stimulated some inflammatory response that was useful for mobilizing stem cells (144). However, excessive infiltration of inflammatory cells may induce apoptosis or necrosis (148). UTMD demonstrably improved angiogenesis, collateral coronary circulation, and myocardial perfusion (144).

The probable mechanism may be that the capillary rupture and the endothelial widening promoted the stem cell attachment to the damaged endothelial layer with cytokine release further enhancing the homing process of the cells (144). The side effects documented for this procedure include necrosis or apoptosis of CMCs, arrhythmias, extensive destruction of the endothelium, and leakage of micro-vessels.

### New Homing Enhancing Technique

Several studies have reported that transplanted EPCs home to regions of ischemic injury (62, 149). However, the precise homing mechanisms are not completely understood; however, playing a central role is the interaction between molecules on the surface of EPCs and upregulated ligands at the site of injury. Recently, Heo et al. (150) showed that homing of transplanted human EPCs and vascular repair was promoted by repeated injections of WKYMVm a synthetic peptide (a chemoattractant for human phagocytes) into ischemic limbs, this subsequently activated formyl peptide receptor 2 (FPR2) on EPCs. EPCs begin to express FPR2 and FPR3 from day 7 to 10 in culture. The binding of WKYMVm to FPR2 induces the proliferating, migrating, and sprouting activities of EPCs (150).

### Stem Cell Modulation

The hunt for the best ways to improve the delivery of stem cells to areas of ischemia appears unending as the fields of immunology, molecular biology, genetic, and proteomics also have their part to contribute. Leading the wave are (i) antibody, (ii) genetic, (iii) selectin, and (iv) peptide-directed approaches (151).

#### Antibodies

These involve two strategies: (i) the use of Palmitate protein-G or A to attach antibodies to the surfaces of cells including stem cells and (ii) the use of antibodies that are “bispecific” having affinities for the cell to be delivered and the region of interest. However, the use of protein-G is more favored as the production of bispecific antibodies could be challenging and have shown some instability. HSCs have been targeted to an injured cardiac vessel using bispecific antibodies; these were done using either anti-c-kit conjugated to anti-VCAM (152), anti-myosin light chain kinase, or anti-CD45 (153).

#### Genetic Manipulation

This is where DNA or RNA is introduced into a cell to aid the expression of ligands on the surface of the cell to enhance homing and attachment to tissue of interest. Increased homing of MSCs transduced with CXCR4 toward an infarcted heart was found to improve cardiac output and angiogenesis within an infarcted heart (154, 155). This method though seems lucrative, but the possibility of its translation for clinical use is certainly daunting.

#### Selectin

This approach is designed to mimic the natural immunity of the body. Sackstein et al. (156) modified the “selectin-directed cell targeting” pioneered by Xia et al. (157) to modify the surface of MSCs forming HCELL, which is a ligand that binds to E-selectin and L-selectin. Stem cells coated with selectin-binding ligands penetrated through the EC and the layers of the basement membrane to migrate to the area of pathology from the circulation.

#### Peptide Directed

Here, stem cells coated with targeting peptides were identified by phage display, the technique was pioneered partly by Kean et al. (151). Tissue or cell ligands can be highly specific, and the cell surfaces targeted with multiple ligands. However, it appears no peptide-targeted therapeutic agent has made it to the market yet about the antibody-based one (158).

### Mannitol-Enhanced Delivery

Adult and neonatal ischemic injury to the brain is quite a clinically significant problem with the paucity of therapeutic interventions. For clinicians at present, the treatment of stroke patients is limited to the use of tPA (159). Stem cell therapy in the brain in small clinical trials has proven to be effective but, however, is limited by their delivery into the injured brain as the brain is protected by a Blood–Brain Barrier (BBB). Surgical delivery though very useful, but it is invasive and is accompanied by its own complications (160). Thus, a minimally invasive approach is more favored. Hence, strategies have been designed to fragment the BBB for the delivered cells to gain entry into the brain substance (159). Gonzales-Portillo et al. did a study et al. which demonstrated that mannitol a substance that transiently opens the BBB-facilitated delivery of stem cells and trophic factors into the brain (159) thus, stem cell therapy combined with mannitol may enhance therapeutic results in adult stroke patients. The use of stem cells in cerebral ischemia has recorded several successes (161, 162), and their combination with mannitol might prove lucrative in the management of stroke patients. However, considering the critical function of the BBB, permeating it might allow harmful or inflammatory chemokines or cytokines to breach the barrier and worsen the pathologic site. Therefore, the use of this technique will put into critical consideration the period of permeation and the size of the leakage for selective passage of stem cells and growth factors.

## Future Work: Combined Delivery of Macrophages and Stem Cells

The process of ischemia results in tissue apoptosis and necrosis which triggers the cleaning up process, angiogenesis, and remodeling. Macrophages have been shown to play an enormous function during apoptosis and necrosis, tissue clean up, repair or remodeling, and angiogenesis in any ischemic event. The increased post-MI myocardial rupture and mortality in subjects who had their immune system suppressed with methylprednisolone in early clinical trials confirmed the decisive role of the inflammatory process influenced primarily by macrophages (163, 164).

The role of macrophages post-ischemic event is critical as it is considered a key player in the post-ischemic regenerative process. Thus, its therapeutic potentials need to be regarded as strongly not just alone as confirmed by the first administration of polarized macrophages by Jetten et al. (39) but possibly in combination with other stem cells for a better therapeutic effect.

*Activation of macrophages* usually occurs on day 3 post-ischemia, and the two main patterns of activation include M1 (classical), activated by LPS or γ-IFN (interferon), and M2 (alternative), which has two subsets M2a and M2c activated by IL-4 and IL-10, respectively. M1 is pro-inflammatory, while M2 is anti-inflammatory (165). See Table 2 below for the summary of secretions.

**Table 2:** **Markers of classical (M1) and alternative (M2) activation**.

Classical (M1)	Alternative (M2)
**Pro-inflammatory**	**Anti-inflammatory**
Fas ligand high	Arginase I/II
Interferon γ high	CD 163
Interleukin-1β high	Fas ligand low
Interleukin-6 high	Basic fibroblast growth factor (FGF)
Interleukin-8 high	Interferon γ low
Interleukin-10 high	Interleukin-4 receptor I
Interleukin-12 high	Interleukin-6 low
Interleukin-23 high	Interleukin-10 high
Matrix metalloproteinases	Interleukin-12 low
Nitric oxide (NO)	Interleukin-23 low
Extracellular matrix destruction	Extracellular matrix reconstruction
Inducible NO synthase	MS-1-high molecular weight protein
Tumor necrosis factor α high	Transforming growth factor β
	Tumor necrosis factor low
	Vascular endothelial growth factor

*Adapted from Ref. (165)*.

Furthermore, macrophage cultures have yielded over 150 secretory proteins secreted by macrophages, and some of these proteins are either pro-angiogenic or angiostatic helping to regulate the angiogenic process tightly. In Table 3 is a few of the key secretory proteins of the macrophage.

**Table 3:** **A few key secretory proteins of macrophages**.

Cytokines and chemokines	Growth factors
α-2 macroglobulin ADAMTS-4, -7, -8, -9 Angiotensin converting enzyme Caspases 2, 3, 8, 9 Cathepsin B Interferon α Interleukin-1 α and β, -6, -8, -10 Macrophage inflammatory proteins MMP-1, -3, -7, -8, -9, -12 Monocyte chemotactic protein-1 Plasmin Plasminogen activator inhibitor 1 Proteases and protease inhibitors Tumor necrosis factor α	Angiotensinogen Basic FGF Endothelial cell inhibitory factor Granulocyte colony stimulating factor Granulocyte-macrophage colony stimulating factor Insulin-like Growth Factor 1 Macrophage colony stimulating factor Monocyte chemotactic protein-1 Substance-P Thrombospondin-1 Transforming growth factors α and β Vascular endothelial growth factor

*Adapted from Ref. (165)*.

A number of macrophages in different injury models such as MI, PAD, stroke, and wound healing have been shown to correlate positively with angiogenesis (166). Macrophages can influence every step of the angiogenic process such as local ECM modification, EC induction to migrate or proliferate, and vascular growth inhibition, once capillaries have been formed (165).

*Macrophage*s induce angiogenesis by expressing MMPs, which disintegrates the basement membrane and degrades the ECM of the capillary, thus facilitating migration of the endothelial cells. Macrophages initially drill a tunnel in the matrix, which is then colonized by either EPCs or capillary sprouts (165). Several MMPs are synthesized by macrophages such as: MMP-1, -3, -7, -8, -9, and -12. However, MMP-9 appears to be most relevant to post-ischemic remodeling, and this is elaborately expressed during the differentiation of macrophages and overexpressed after an ischemic event (167). Macrophages also release (TIMPs) which functions to inhibit MMPs as well regulating cell growth and signaling (168).

The fact that tissue ischemia results in reduced blood flow limit the number of monocytes that are available to differentiate into macrophages at the region of ischemia, hence the benefit of the exogenously delivered polarized macrophages. Jetten et al. demonstrated that both M1 and M2 macrophages act in a complementary manner to stimulate arteriogenesis. M1 pro-angiogenic factors, TNF-α and iNOS, and M2-FGF-2 worked in concert with other macrophage-derived growth factors and cytokines during vascular remodeling. Whereas, the non-polarized macrophages did not demonstrate any collateral flow. A possible downside to macrophage delivery might be tissue inflammation; however, there was no documentation of this in the study of Jetten and colleagues. This might be due to the number of the macrophages delivered in the study or the opposing effect of the anti-inflammatory M2 cytokines TGF-B and IL-10 on M1. Hence, further studies might be required to investigate possible tissue inflammation.

**Figure 7:**
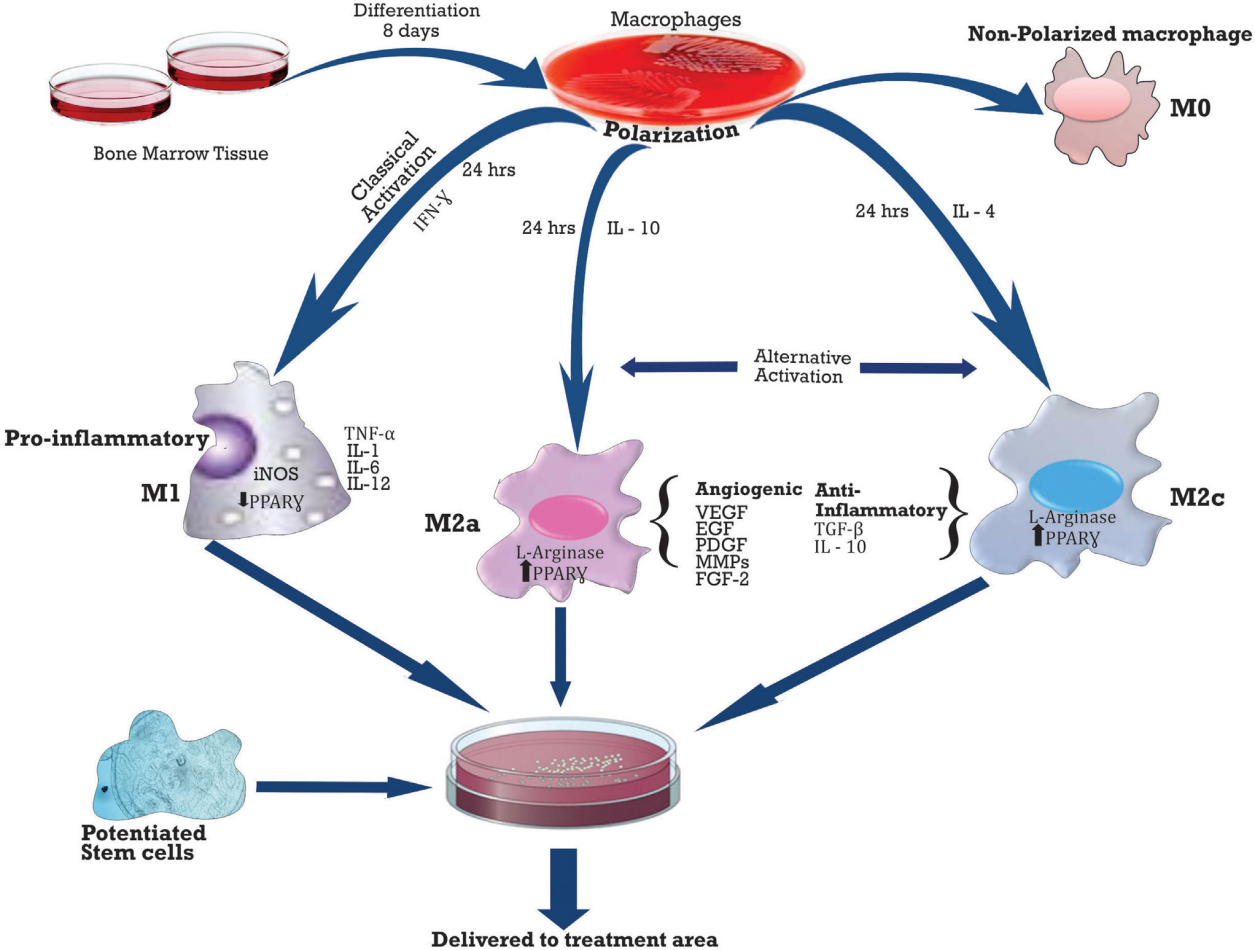
**Delivery of Polarized macrophages with potentiated stem cells**.

Since the goal of delivering stem cells is to achieve the most efficient vascular and tissue regeneration, the tissue reparative role of the macrophage, however, impressive, might not be sufficient. Therefore, studies might be designed to harness the possible synergistic benefits of combining polarized macrophages with potentiated stem cells and delivered in the most efficient way to the region of ischemia. See Figure 7 below.

## Conclusion

This study reviewed quite a variety of available new methods of delivering stem cells. A number of these new methods minimized the invasiveness of delivering stem cells to the subject. These delivery methods resulted in improved retention, survival, and engraftment of these cells and led to remarkable improvements in angiogenesis, collateral vessel formation, improved tissue perfusion, and ultimately salvaged and significantly restored tissue function. Though these studies yielded good success rates in animal models, they however still require extensive studies for translation into humans. The study also proposed possible synergistic benefits that might exist in the efficient delivery of a combination therapy of polarized macrophages with stem cells as this might enhance not only angiogenesis but an efficiently regenerated tissue.

## Author Contributions

AF contributed solely to this review with the exception of those listed in the acknowledgement.

## Conflict of Interest Statement

The author declares that the research was conducted in the absence of any commercial or financial relationships that could be construed as a potential conflict of interest.
